# The use of digital PCR to improve the application of quantitative molecular diagnostic methods for tuberculosis

**DOI:** 10.1186/s12879-016-1696-7

**Published:** 2016-08-03

**Authors:** Alison S. Devonshire, Denise M. O’Sullivan, Isobella Honeyborne, Gerwyn Jones, Maria Karczmarczyk, Jernej Pavšič, Alice Gutteridge, Mojca Milavec, Pablo Mendoza, Heinz Schimmel, Fran Van Heuverswyn, Rebecca Gorton, Daniela Maria Cirillo, Emanuele Borroni, Kathryn Harris, Marinus Barnard, Anthenette Heydenrych, Norah Ndusilo, Carole L. Wallis, Keshree Pillay, Thomas Barry, Kate Reddington, Elvira Richter, Erkan Mozioğlu, Sema Akyürek, Burhanettin Yalçınkaya, Muslum Akgoz, Jana Žel, Carole A. Foy, Timothy D. McHugh, Jim F. Huggett

**Affiliations:** 1 Molecular and Cell Biology, LGC, Queens Road, Teddington, TW11 0LY UK; 2 Centre for Clinical Microbiology, Department of Infection, University College London, Royal Free Campus, London, UK; 3 European Commission, Joint Research Centre (JRC), Institute for Reference Materials and Measurements (IRMM), Geel, Belgium; 4 National Institute of Biology, Večna pot 111, 1000 Ljubljana, Slovenia; 5 Vircell S.L. Molecular Diagnostic Department, The Technology Park of Health Sciences, Granada, Spain; 6 TB Supranational Reference Laboratory, San Raffaele Scientific Institute, Milan, Italy; 7 Microbiology, Virology and Infection Control, Great Ormond Street Hospital NHS Foundation Trust, London, UK; 8 Centre for Clinical Tuberculosis Research, TASK Applied Science, Cape Town, South Africa; 9 SA MRC Centre for TB Research, DST/NRF Centre of Excellence for Biomedical Tuberculosis Research, Division of Molecular Biology and Human Genetics, Faculty of Medicine and Health Sciences - Stellenbosch University, Francie van Zijl Drive, Tygerberg 7505, PO Box 241, Cape Town, 8000 South Africa; 10 KCRI, Moshi, Tanzania; 11 BARC-SA and Mycobacteriology Department, Lancet Laboratories, Johannesburg, South Africa; 12 Nucleic Acid Diagnostics Research Laboratory (NADRL), Microbiology, School of Natural Sciences, National University of Ireland, Galway, Ireland; 13 Forschungszentrum Borstel, National Reference Centre for Mycobacteria, Borstel, D-23845 Germany; 14 UME, Ulusal Metroloji Enstitüsü, Gebze, Kocaeli Turkey; 15 School of Biosciences & Medicine, Faculty of Health & Medical Sciences, University of Surrey, Guildford, UK

**Keywords:** Digital PCR, Diagnostics, *Mycobacterium tuberculosis*

## Abstract

**Background:**

Real-time PCR (qPCR) based methods, such as the Xpert MTB/RIF, are increasingly being used to diagnose tuberculosis (TB). While qualitative methods are adequate for diagnosis, the therapeutic monitoring of TB patients requires quantitative methods currently performed using smear microscopy. The potential use of quantitative molecular measurements for therapeutic monitoring has been investigated but findings have been variable and inconclusive. The lack of an adequate reference method and reference materials is a barrier to understanding the source of such disagreement. Digital PCR (dPCR) offers the potential for an accurate method for quantification of specific DNA sequences in reference materials which can be used to evaluate quantitative molecular methods for TB treatment monitoring.

**Methods:**

To assess a novel approach for the development of quality assurance materials we used dPCR to quantify specific DNA sequences in a range of prototype reference materials and evaluated accuracy between different laboratories and instruments. The materials were then also used to evaluate the quantitative performance of qPCR and Xpert MTB/RIF in eight clinical testing laboratories.

**Results:**

dPCR was found to provide results in good agreement with the other methods tested and to be highly reproducible between laboratories without calibration even when using different instruments. When the reference materials were analysed with qPCR and Xpert MTB/RIF by clinical laboratories, all laboratories were able to correctly rank the reference materials according to concentration, however there was a marked difference in the measured magnitude.

**Conclusions:**

TB is a disease where the quantification of the pathogen could lead to better patient management and qPCR methods offer the potential to rapidly perform such analysis. However, our findings suggest that when precisely characterised materials are used to evaluate qPCR methods, the measurement result variation is too high to determine whether molecular quantification of *Mycobacterium tuberculosis* would provide a clinically useful readout. The methods described in this study provide a means by which the technical performance of quantitative molecular methods can be evaluated independently of clinical variability to improve accuracy of measurement results. These will assist in ultimately increasing the likelihood that such approaches could be used to improve patient management of TB.

**Electronic supplementary material:**

The online version of this article (doi:10.1186/s12879-016-1696-7) contains supplementary material, which is available to authorized users.

## Background

Using molecular methods, such as the polymerase chain reaction (PCR), to diagnose tuberculosis (TB) offers the potential for a simple, rapid and objective alternative to microbial culture and smear microscopy. While there have been a range of commercially available tuberculosis molecular diagnostic tests for over 15 years [1] it is the introduction of the Cepheid Xpert® MTB/RIF test (Xpert MTB/RIF) [2], and its subsequent recommendation by the WHO [3], that has changed how molecular based methods are viewed as tools that could impact on the management of this global disease. As a consequence, the Xpert MTB/RIF has been rolled out in a number of countries in the developing world [4] and is the most commonly used direct molecular test in countries like the United Kingdom [5].

As molecular diagnosis of TB becomes more widespread there is the need to support the routine application of such methods through the development and application of reference materials for the calibration of in vitro diagnostics and external quality assessment (EQA), both of which are scarce. There is often a regulatory requirement to use EQA so it is vital that proper quality controlled methods are developed. The proper calibration of such in vitro diagnostics is in many places, such as the European Union where the Directive on in vitro diagnostic medical devices 98/79/EC applies, a requirement. A limited portfolio of nucleic acid and bacterial extract materials has been developed as quality assurance controls for molecular diagnosis of tuberculosis [6, 7]. These have been prepared and characterised using a variety of methods including colony forming units (CFU) counting and real-time quantitative PCR (qPCR), to support methods like the Xpert MTB/RIF. These reference standards are important developments as they enable laboratories to compare the performance of various TB diagnostic tests performed in house, while also facilitating comparisons of the associated diagnostic services offered nationally and eventually between countries. However, to have the greatest impact, these materials should be evaluated with an independent method with a high level of accuracy to enable a judgement about the equivalence of measurement results obtained with materials from different EQA providers, or even batches from the same scheme. Such reference methods are common in clinical biochemical measurement [8], however to date do not exist for molecular microbiological measurement.

While TB is diagnosed using a qualitative approach, with clinical decisions made on presence or absence of the pathogen, it also represents an example where pathogen quantification is desirable as a potential indicator to determine treatment efficacy and predict relapse. Quantification is currently applied in patient management by grading of smear positivity and there are a number of studies that have used more sophisticated culture based quantitative metrics such as CFU counting and time to positivity [9, 10] as well a molecular based methods targeting RNA [11, 12] and DNA using PCR [13, 14] and, increasingly, qPCR using the Xpert MTB/RIF [15–18]. Where the quantitative capabilities of the Xpert MTB/RIF have been evaluated its value as a quantitative method is equivocal. In some studies it correlated with other methods in pulmonary TB [15, 17, 19] whereas in others it was found not to agree with culture methods reported as being superior [16].

To resolve such disagreement, and ultimately determine the efficacy of quantitative molecular measurement using any method, a metrological approach can be adopted and analytical and biological sources of variation defined. Defining the former measurement uncertainty [20] allows improvements to be made on the analysis method, while the latter provides information on what is clinically possible. To accurately determine the error and define the quantitative performance of a given method across different laboratories, rigorously characterised materials are required of which property values have been determined using a high accuracy method [21]. Digital PCR (dPCR) [22, 23] may offer such an approach through binary counting of the number of pathogen genomes present which does not rely on an additional calibration material like qPCR, and has been successfully used in the quantification of a range of microorganisms [24–29]. dPCR is more precise than qPCR [30] and offers a potentially accurate method for quantifying pathogens in reference materials when coupled with an efficient extraction protocol [24]. In dPCR a limiting dilution of the template is initially performed into many small volume partitions prior to running the PCR reactions. Following the reaction quantification is achieved by measuring partitions as either negative (without template) or positive (with template). In this study we investigated the accuracy (comprising sources of bias and precision) of dPCR for quantifying sequence-specific DNA concentration in EQA materials that were then used to characterise the qualitative and quantitative performance of molecular diagnostic testing across eight different clinical laboratories applying qPCR and Xpert MTB/RIF.

## Methods

Three different experimental comparisions were performed in the study to assess the performance of dPCR when measuring *M. tuberculosis* in terms of 1) quantification, 2) reproducibility and 3) performance in supporting molecular quantification using routine clinical tests.

### Study materials

To enable the characterisation of dPCR reproducibility and to determine sources of error, three different types of reference materials were investigated comprising gene fragments cloned into a plasmid, genomic DNA (gDNA) and whole mycobacteria in synthetic sputum [31].

To assess dPCR quantification reproducibility when measuring the gene targets without the presence of a large more complex genome, a ‘TB Control plasmid’ was used [24]. This consisted of a pUC19 plasmid containing an insert including 16S rRNA and *rpoB* genes from *M. tuberculosis*. The linearised plasmid (Additional file 1: Method 1) was gravimetrically diluted to ~10^5^ copies/μL, in a DNA carrier of sonicated human gDNA (Cambio) diluted to 25 ng/μL in 1× TE pH 8.0 (Ambion), as described in the Additional file 2. Two hundred × 50 μL units of the diluted plasmid were added into low DNA binding microcentrifuge tubes and stored at −20 °C.

To assess dPCR quantification reproducibility when measuring targets using gDNA, a material comprising a commercial preparation of gDNA from *M. tuberculosis* (Zopf) Lehmann and Neumann (ATCC® 25618™), was prepared (H37Rv gDNA). The gDNA was initially quantified by fluorimetry using a dsDNA BR assay (Qubit, *Thermo Fisher Scientific*), and was subsequently gravimetrically diluted to approximately 10^5^ copies/μL in 1× TE pH 8.0 (Ambion) containing ~25 ng/μL sonicated human genomic DNA. Two hundred units consisting of ~50 μL diluted gDNA were produced in low DNA binding tubes and stored at −20 °C.

To assess reproducibility of dPCR measurements, when including the extraction step necessary for clinical analysis, two additional materials were used:

A) 300 units of a whole mycobacterial panel in artificial sputum (BCG/ASM) were prepared. *Mycobacterium bovis* BCG strain Pasteur (BCG) was grown in 7H9 liquid medium (Becton-Dickinson (BD)) containing 10 % albumin-dextrose-catalase (ADC) (BD) enrichment medium and 0.2 % Tween 80. CFU (mean 1.09 × 10^8^ ± SD 1.98 × 10^7^ per mL) was determined using the Miles and Misra method [32] and nine serial dilutions of the stock culture were plated onto 7H10 solid agar (BD) containing 10 % OADC (oleic acid-albumin-dextrose-catalase, BD) and 0.5 % glyercol. Artificial sputum matrix (ASM) was prepared according to Dinesh [31]. Each unit of BCG/ASM material was prepared containing 100 μL BCG suspension and 900 μL ASM. Aliquots were centrifuged 18,000 × *g* for 10 min and bacterial pellets were resuspended in 400 μL 1 × TE buffer followed by heat treatment for 30 min at 95 °C and frozen at −80 °C.
B) For the clinical laboratory comparison an additional whole mycobacterial material preparation was used. ‘Total MTB Control’ was supplied by Vircell (Santa Fe, Spain) as AMPLIRUN® Total MTB Control (Sputum); product reference MTBC013.

### DNA extraction

For assessment of dPCR when measuring whole mycobacteria, DNA was extracted from BCG/ASM and Total MTB Control materials using the Cetyltrimethylammonium bromide (CTAB) NaCl method [33] as described in Addititonal file 1 with a final resuspension volume of 200 μL. For gDNA extraction from the Total MTB Control, the lyophilised material was initially reconstituted in 400 μL nuclease free water (Ambion) and the final pellet was resuspended in 50 μL TE buffer (Fluka).

For the comparision of qPCR by the clinical laboratoraties three specified extraction protocols were followed (details in Additional file 1: Table S4).

### dPCR method

Primers/hydrolysis probes (Additional file 1: Table S1) used for quantification of 16S rRNA and *rpoB* genome copies were as described [12, 24]. dPCR was performed on the Biomark HD System for Genetic Analysis (Fluidigm, San Francisco, CA) using 48-panel 37 K dPCR integrated fluidic circuits, the QuantStudio® 3D Digital PCR System using QuantStudio® 3D Digital PCR Chips (ThermoFisher Scienific) or the QX100 Droplet Digital PCR System (Bio-Rad) with respective reaction mixes as per Additional file 2 and thermal cycling parameters as per Additional file 1: Table S2. The concentrations, termed λ (also known copies per partition), of the different templates added to the different dPCR reactions are described in Additional file 1: Table S3. The dMIQE (Minimum Information for publication of Quantitative Digital PCR Experiments) checklist for this study can be found in Additional file 3.

### Characterisation of panels

#### Homogeneity

All homogeneity analysis was performed using the Biomark HD System for Genetic Analysis applying the assay targeting *rpoB*. The variations between ten randomly selected replicate units, each of all four types of study materials were determined in order to assess the homogeneity of the materials. For each unit of TB Control Plasmid and H37Rv gDNA materials, four dPCR replicates were performed. gDNA was extracted from each unit of BCG/ASM and Total MTB Control whole microbe materials using the CTAB/NaCl method described above. Each BCG/ASM gDNA extract was diluted 1:10 in nuclease free water (Ambion) containing sonicated human gDNA (25 ng/μL) (Cambio) prior to analysis of 2 μL template by Biomark HD dPCR (*n* = 4). Each Total MTB Control gDNA extract was analysed without dilution by Biomark HD dPCR (*n* = 4) using 3 μL template.

#### Stability testing

Short term stability testing (dry ice, 4 °C and 40 °C for 7 and 14 days) was performed to simulate the effect of transport. Details of the protocols can be found in Additional file 1: method 3.

### Reproducibility of dPCR

The reproducibility of a candidate dPCR-based reference method for quantification of *M. tuberculosis* was assessed through an inter-laboratory study involving four metrology institutes each performing the *rpoB* assay used in defining the material homogeneity and the 16S rRNA assay. Plasmid and gDNA materials were analysed using three dPCR platforms: two chip-based (Biomark (Fluidigm) and Quantstudio 3D (Thermo Fisher Scientific) and one droplet-based (QX100, Bio-Rad). Three of the four laboratories also performed gDNA extraction from the BCG/ASM material and analysed extracts using the Biomark HD and QX100 platforms. Protocols for gDNA extraction and dPCR were provided as per Additional file 2; the protocol for the Quantstudio 3D method can be found in Additional file 1: method 2.

### End user analysis by clinical laboratories

To assess the role of the rigorously characterised prototype reference materials the BCG/ASM and Total MTB Control materials were used to evaluate the quantitative performance of eight different end user laboratories (Additional file 1: Tables S4 and S5).

### qPCR

Three laboratories received three units of the BCG/ASM and Total MTB Control materials for analysis using in-house developed gDNA extraction and qPCR protocols (Additional file 1: Table S4). One laboratory performed both qPCR and Cepheid Xpert MTB/RIF analysis. Laboratory identifiers were removed and replaced with greek numerals for the purpose of the qPCR comparison.

### Xpert MTB/RIF

Six laboratories received three units of the BCG/ASM and Total MTB Control materials for analysis using the Xpert MTB/RIF assay (Cepheid) (respective details in Additional file 1: Table S5). Prior to processing, the Total MTB Control material was re-suspended in 1.0 mL of molecular biology grade water (Ambion) and mixed until completely reconstituted. Following thawing, molecular biology grade water (0.6 mL) was added to the BCG/ASM material to achieve a final volume of 1.0 mL for all test materials. Laboratories were instructed to process the test materials as per the manufacturer’s instructions for an expectorated sputum sample using the Xpert MTB/RIF test. Laboratory identifiers were removed and replaced with numbers for the purpose of the Xpert MTB/RIF comparison.

### Data analysis

Data from the characterisation of 10 units of each study material in terms of homogeneity was analysed in R version 3.0.1 in order to determine the single laboratory-assigned values. A mixed effect model was fitted with sample unit as a random effect and the mean as the single fixed effect. The model was fitted for concentration, for which the residuals were sufficiently normally distributed. The means, standard errors of the means and homogeneity uncertainties (between unit standard deviations) were calculated.

Data from the dPCR inter-laboratory study were used to calculate concentration values and measurement uncertainties for the TB Control Plasmid, H37Rv gDNA and BCG/ASM based on the approach of the assignment of indicative copy number concentration values by dPCR used by JRC-IRMM for reference material ERM-AD442k [34]. Mean values for each dataset combining laboratory and dPCR platform (for example, Laboratory 1/Biomark) were calculated from all replicates. Copy number concentration values for the materials were calculated from the mean and standard error of the six (TB Control Plasmid, H37Rv) or five (BCG/ASM) mean values of the respective laboratory platforms. The expanded uncertainty was calculated by applying a coverage factor (*k*) to the standard error at the 95 % confidence level based on the number of degrees of freedom associated with the number of laboratory/platform datasets (*k* = 2.57 for plasmid and H37Rv gDNA, and *k* = 2.78 for BCG/ASM).

For the end user clinical analysis differences in measurements of BCG/ASM and Total MTB Control results were assessed by subtracting the average Cq of the BCG/ASM from the Total MTB Control and the delta Cq (ΔCq) was determined. For the Xpert MTB/RIF the average Cq from the five probes was taken for each analysis. Fold differences were calculated using the following equation:

$$ Fold\  difference = {2}^{\Delta Cq} $$

## Results and discusssion

### Quantification of materials

In the initial stage of this study we assessed dPCR as a method to quantify specific DNA sequences in different prototype reference materials and assess their stability and homogeneity to support proficiency testing of clinical molecular analysis of *M. tuberculosis*. Three types of materials were prepared consisting of different levels of complexity: (i) linearised ‘TB Control Plasmid’ DNA containing the full length *rpoB* and 16S rRNA genes; (ii) purified gDNA from *M. tuberculosis* laboratory reference strain H37Rv and (iii) two preparations of whole mycobacteria in synthetic sputum: BCG/ASM material and Total MTB Control.

Certified reference materials of defined homogeneity have been made available for molecular testing of human cytomegalovirus [35] and BCR-ABL [36]. However, this has to our knowledge not been done with the intention to support molecular testing of bacteria. Establishing reference material homogeneity and stability is a prerequisite to determine measurement precision within and between laboratories, which is ultimately necessary for robust quantification. This is in recognition of the fact that different units of a batch of reference materials can, and often do, contain different amounts of the component being measured. These factors can, if necessary, be mathematically considered for separating different sources of measurement result variability.

**Fig. 1:**
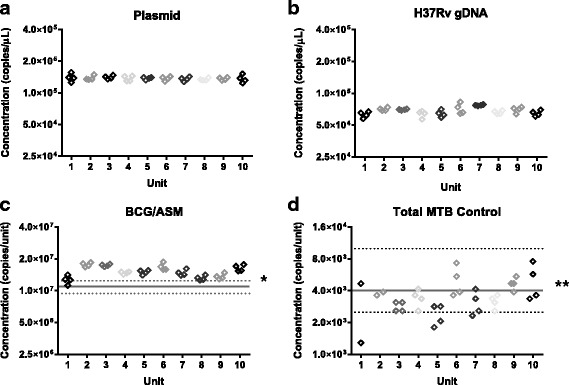
Homogeneity assessment of ten randomly selected tubes of (**a**) TB Control Plasmid (**b**) H37Rv gDNA, (**c**) BCG/ASM and (**d**) Total MTB control. * solid line depicts mean value and 95 % CI measured using colony forming units. ** solid line depicts value and range provided by the product sheet

In this study 10 randomly selected units of each material were used to characterise the material homogeneity (Fig. 1) and stability (Additional file 1: Figure S1). For the TB Control Plasmid material, no significant difference in DNA concentration between units was observed. Therefore, no allowance for homogeneity was included in the value assignment of this material (Table 1). For the H37Rv gDNA, BCG/ASM and Total MTB Control Materials a contribution for homogeneity was included in describing the respective uncertainties (Table 1) due to differences between units (Fig. 1). Expanded uncertainties were calculated by applying a coverage factor (*k*) based on 9° of freedom for H37Rv gDNA, BCG/ASM and Total MTB Control materials (*k* = 2.26) and 30° of freedom for the Plasmid DNA material (*k* = 2.04) as the between unit variability was larger than residual technical (dPCR) variability for the H37Rv gDNA, BCG/ASM and Total MTB Control materials, while for the TB Control Plasmid the between unit variability was negligible.

**Table 1:** Description of test materials

Material	LGC-assigned value		Inter laboratory study assigned value	
	Value	Expanded measurement uncertainty^a^	Value	Expanded measurement uncertainty^a^
Plasmid(copies/μL)	1.374 × 10^5^	2.3 × 10^3^	1.30 × 10^5^	1.5 × 10^4^
H37Rv gDNA(copies/µL)	6.83 × 10^4^	3.4 × 10^3^	4.8 × 10^4^	9.0 × 10^3^
BCG/ASM(copies/unit)	1.52 × 10^7^	1.3 × 10^6^	1.24 × 10^7^	2.4 × 10^6^
Total MTB control(copies/unit)	3.47 × 10^3^	6.6 × 10^2^	N/A	N/A

^a^Measurement uncertainty rounded outwards to 2 s.f. Concentration values given to the same integer as measurement uncertainties

Although we can detect quantitative differences between units by performing this assessment, we were able to demonstrate that these differences are small and the assessment provides the basis on which the preparation of these materials can be improved.

The process of analysing the whole cell TB materials also enabled us to assess dPCR in terms of accuracy, as already performed for extracted DNA [24]. When compared to CFU counting, dPCR provided a very similar result demonstrating that both methods can deliver equivalent results (Fig. 1c). This suggested that, when coupled with an efficient extraction method, dPCR can offer a complementary method to CFU counting which is faster and, as it measures DNA, is potentially more suitable for characterising reference materials for molecular diagnostic tests like the Xpert MTB/RIF.

### Assessment of the reproducibility of the dPCR method

**Fig. 2:**
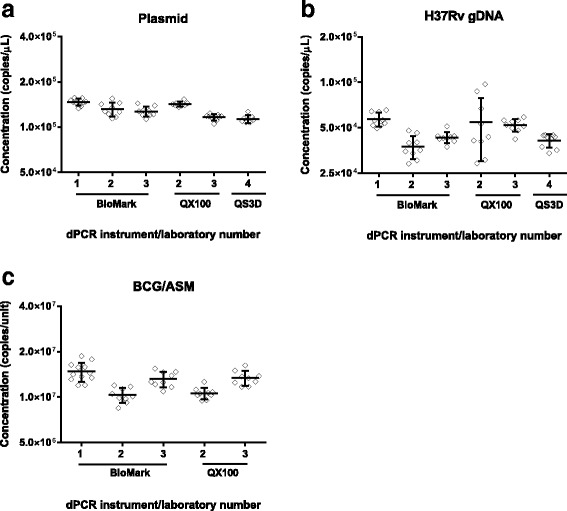
Assessment of the reproducibility of dPCR measuring (**a**) TB Control Plasmid and (**b**) H37Rv gDNA with four laboratories and three different dPCR instruments or (**c**) BCG/ASM three laboratories and two different dPCR instruments

While dPCR may be able to provide equivalent results to CFU counting in a single laboratory, for maximum impact this must be the case with a satisfactory reproducibility across multiple laboratories, ideally with different instruments. In the next stage of the study the two DNA materials and the BCG/ASM whole mycobacterial material were used to evaluate the reproducibility of the dPCR method by comparing the results from three or four different laboratories using two or three different dPCR instruments (Fig. 2). ANOVA analysis demonstrated a difference between laboratories when the TB Control Plasmid was measured (*p* = <0.001). As this is the least complex template investigated with the highest intra-laboratory precision, this result suggested effects from differences in instrument and/or laboratory setups (Fig. 2a). Larger variations were observed for within- and between- laboratory results for the H37Rv gDNA (Fig. 2b) compared to TB Control Plasmid. This may be attributable to the more complex secondary structure of gDNA and its impact on dPCR amplification [37, 38]. However, the fact that differences in the results for plasmid and gDNA were not instrument specific suggests that this is likely to be due to laboratory setups rather than the choice of instruments. Furthermore, even with the most complex whole cell material, the measured differences were less than two fold (Fig. 2c).

Concentration values and measurement uncertainties using the mean values for each combination of laboratory and platform were also calculated for the TB Control Plasmid, H37Rv gDNA and BCG/ASM materials (Table 1). The mean values calculated from the inter-laboratory study were within 30 % of those initially calculated by the coordinating laboratory (TB Control Plasmid, 95 %; H37Rv gDNA, 70 % and BCG/ASM, 82 %). The measurement uncertainties estimated for the inter-laboratory dPCR comparison study were larger compared to those from the homogeneity study performed at a single laboratory (expressed relative to the concentration value): TB Control Plasmid, 12 % vs. 1.7 %; H37Rv gDNA, 19 % vs. 5.0 % and BCG/ASM, 19 % vs. 8.5 %. This reflects the additional sources of measurement error such as dPCR platform and differences in execution of detection and extraction protocols which are taken into account with inter-laboratory comparisons.

These findings demonstrate that while there are differences between laboratory, as seen in Fig. 2, these are small (being less than two fold). As the interlaboratory reproducibility was predicted to be greater than this we therefore concluded that the reproducibility of the dPCR method was satisfactory for the remainder of this study. These findings complement the precision and potential trueness already described elsewhere [24, 39]. As with the homogeneity data, these results provide a key reference point on which we can build on to better understand sources of bias when measuring *M. tuberculosis* using molecular methods. This can be used to further improve reproducibility, which can in turn improve the performance of quantitative molecular measurements associated with TB diagnostic and prognostic monitoring. In the short term, this could be achieved by using dPCR as a reference method to quantify pathogens in reference materials. These could in turn support more routine application of other molecular methods, like qPCR, when measuring *M. tuberculosis* and other pathogens. Ultimately the findings presented here make a case for dPCR to be explored and developed as a quantitative molecular diagnostic test for diseases like TB.

### Assessment of clinical laboratories using quantitative molecular methods

In the final part of the study we assessed how the comprehensively characterised BCG/ASM and Total MTB Control whole mycobacteria prototype reference materials could be used to compare routine molecular diagnostic laboratories performance using either qPCR or Xpert RIF/MTB. As both methods also provide a quantitative output we wanted to use these materials to explore the measurement reproducibility of the clinically applied molecular methods independent of clinical/biological factors.

The comparison of results showed that all laboratories were able to determine that the BCG/ASM and Total MTB Control materials were relatively high and low, respectively, in *M. tuberculosis* abundance independent of the chosen method (Additional file 1: Figure S2). When using the Xpert MTB/RIF there was a discrepancy between the levels of bacteria reported (Additional file 1: Figure S2). This was comparable to the findings from an earlier study [40] with quantification cycle (Cq [41] also known as Ct) standard deviations being less than two cycles (Additional file 1: Figure S2c).

A problem when performing qPCR, either using an a laboratory developed method or using the Xpert MTB/RIF, is that the output Cq is a value that should not be directly compared between different methods (qPCR protocols and Xpert MTB/RIF). This is because Cq can be influenced by a range of factors including threshold setting, probe choice and instrument. It is likely that the Cq value are more comparable between different GeneXpert instruments than with qPCR instruments. However, as this parameter is also influenced by amplification curve performance and fluorescence background, a direct comparison of values should be approached with caution.

**Fig. 3:**
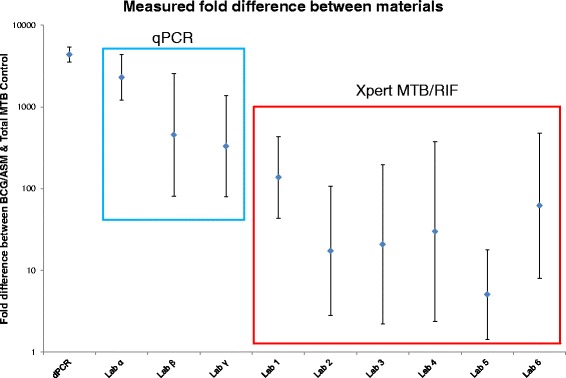
Fold difference between BCG/ASM and Total MTB control when measured using dPCR and compared to eight clinical testing laboratories applying qPCR or Xpert MTB/RIF

What can be directly compared when using qPCR are the differences between Cq values (or ΔCq) from the same assays measured by the same instrument [41]. Using the results from the clinical testing laboratories, we conducted a comparison between results on the BCG/ASM and Total MTB Control materials which when measured using dPCR differ by a factor of ~1000. To compare this by Xpert MTB/RIF, we used previous work by Blakemore et al. [42] and later by van Zyl-Smit [18] who suggested that the PCR efficiency of the Xpert MTB/RIF approximates 100 %. Consequently a difference of 2 Cq corresponds to a fourfold change. Of note is that this approach must be used with caution as efficiency will define the accuracy of the fold change estimation [43]. In this case such a comparison demonstrated that many of the clinical laboratories underestimated the difference between the two materials (Fig. 3). Any reduction in PCR efficiency would have further reduced the measured fold difference all of which underestimate the difference determined by dPCR (i.e. Fig. 3 presents the best case scenario). Variation in qPCR efficiency could explain why the variability is large for many of the results, which supports the need to determine efficiency for precise quantification when using qPCR.

The results presented in Fig. 3 clearly demonstrate that the magnitude of the measured fold difference between materials can change significantly between methods. This is perhaps not surprising for the qPCR methods as they use a variety of extraction and PCR methods and do not have access to common reference materials to harmonise their findings. Where molecular quantification has been established in the field of clinical virology, large differences are experienced as the norm when performing qPCR without access to reference materials [44].

It is noteworthy, considering how much work has been performed assessing the Xpert MTB/RIF for quantification [15–17, 19, 45], that based on these findings speculation that such an approach used clinically to quantify the pathogen load in a patient may be premature. At first glance the observed bias leading to reduced fold difference estimates could be explained by the fact that the BCG/ASM concentration is at the upper working range of the Xpert RIF/MTB, however the distribution of MTB detection level results of the BCG/ASM suggests that this was not the cause (Additional file 1: Figure S2a). Neither Cepheid nor the WHO recommend the Xpert MTB/RIF for quantification and we support this view.

While the nature of qPCR means quantification is possible, our findings suggest accuracy and reproducibility need to be assessed and improved before this method can be used to provide the clinical feasibility of molecular quantification of *M. tuberculosis* during the management of TB. Reference materials, such as those described here and by others [6], are necessary to assist in assessing laboratory proficiency in routine testing. However, we demonstrate they also provide a mechanism by which the reproducibility of molecular methods can be evaluated during the translational stage of method development. Furthermore, dPCR offers a method with sufficient accuracy to quantify specific DNA sequences in reference materials for calibration and assessment of the performance of clinical laboratories conducting molecular analysis to assist in the treatment of TB.

## Conclusion

Quantification is increasingly proposed as a tool to assist in the management of patients with bacterial infections such as TB. However, molecular methods that have not been validated for quantification, such as the Xpert RIF/MTB, should be used with caution for this purpose. The findings of this study clearly demonstrate how rigorously characterised reference materials can be used to evaluate within and between laboratory performance. Furthermore dPCR offers a sufficiently accurate method by which these materials can be characterised. What we did not assess in this study was the impact of factors such as different laboratory workers, reagent lot and storage, and it is possible that controlling these factors could improve the findings. Reference materials would be crucial to such an effort as they can be used to define and reduce analytical variation and improve method performance to ultimately determine if quantification of a given pathogen in a clinical context is possible. The high accuracy of dPCR also makes this approach potentially a more robust pathogen quantification technique for clinical purposes in future patient management.

## Abbreviations

ADC, albumin dextrose catalase; ASM, artificial sputum matrix; CFU, colony forming units; Cq, quantification cycle; CTAB, cetyltrimethylammonium bromide; dPCR, digital PCR; EQA, external quality assessment; gDNA, genomic DNA; OADC, oleic acid albumin dextrose catalase; qPCR, quantitative PCR

## Additional files

Additional file 1: Method for restriction digestion of the TB Control Plasmid, dPCR analysis using the QuantStudio 3D platform, dPCR primers and thermal cycling conditions information, dPCR partition volume and partition number and mean λ values. Results from stability testing of the materials and analysis of BCG/ASM and Total MTB Control materials by Xpert MTB/RIF end-user laboratories including instrument and assay information, extraction and qPCR methods used by qPCR end user laboratories and their reported Cq values. (DOCX 114 kb) Additional file 2: Protocol for gDNA extraction and dPCR quantification of materials. (PDF 394 kb) Additional file 3: The dMIQE checklist for this study. (XLSX 18 kb)
