# Supplementary material for: The use of digital PCR to improve the application of quantitative molecular diagnostic methods for tuberculosis
Source: BMC Infect Dis. 2016 Aug 3;16:366. doi: 10.1186/s12879-016-1696-7 (PMC4971652; doi:10.1186/s12879-016-1696-7)
Supplement: Additional file 1: — Method for restriction digestion of the TB Control Plasmid, dPCR analysis using the QuantStudio 3D platform, dPCR primers and thermal cycling conditions information, dPCR partition volume and partition number and mean λ values. Results from stability testing of the materials and analysis of BCG/ASM and Total MTB Control materials by Xpert MTB/RIF end-user laboratories including instrument and assay information, extraction and qPCR methods used by qPCR end user laboratories and their reported Cq values. (DOCX 114 kb) [file 12879_2016_1696_MOESM1_ESM.docx]

**Additional File 1**

**Method 1: Restriction digestion of the TB Control Plasmid**

Approximately 100 ng of the control plasmid was linearised with 10 Units of HindIII restriction enzyme (New England Biolabs), in a final reaction volume of 50 µL. The restriction digestion was carried out at 37 °C for 3 hours, followed by a 20 minute heat deactivation at 80 °C. Linearisation was confirmed by microfluidic capillary electrophoresis using the Agilent Bioanalyzer in conjunction with a DNA 12000 assay. Quantification of the linearised plasmid was performed using a Qubit 2.0 fluorometer with a High sensitivity DNA Kit (Thermo Fisher Scientific).

**Method 2: dPCR analysis using QuantStudio 3D platform (NMI inter-laboratory study)**

The following instrumentation (Life Technologies) was used for analysis using the QuantStudio 3D (former Life Technologies, present supplier is Thermo Fisher Scientific; the following catalog numbers are given from Life Technologies): Dual Flat Block GeneAmp® PCR System 9700 (Cat# 4486414) and QuantStudio™ 3D Digital PCR Chip Loader (Cat# 4482592). The following materials/reagents were used according to the manufacturer’s instructions: QuantStudio™ 3D Digital PCR Chip (Cat# 4485507 for v1, as the new version of the chips were released as version 2), QuantStudio™ 3D Digital PCR 20K Chip Spares Kit (Cat#4485510), QuantStudio™ 12K FlexOpenArray® Immersion Fluid and UV-Activated Chip Sealant Syringe (Cat# 4484475) and QuantStudio™ 3D Digital PCR Master Mix (Cat#4482710 for v1).

Three independent experiments were performed with a single rpoB or 16S rRNA dPCR assay (chip) for each unit of study material per experiment (compared to 2 for BioMark and QX100 dPCR users). Each 15 μL assay contained 3 μL template sample diluted gravimetrically according to the protocol in Supplementary Information 1. Two to three readings were performed for each chip using the QuantStudio™ 3D Digital PCR Instrument. Filled wells and negative wells were identified by setting a quality threshold of 0.5. The fluorescence threshold was set manually. The number of qualified wells, number of negative wells and DNA copies/μL of the reaction were reported.

**Method 3: Short term stability testing**

Short term stability of the BCG/ASM, plasmid and *M. tuberculosis* H37Rv genomic gDNA materials was evaluated by storing three replicate vials of each material at each of three storage conditions/ durations (dry ice, 4 °C and 40 °C for 7 and 14 days). To ensure that the storage terms concluded simultaneously, ‘seven day’ samples were stored seven days after the corresponding ‘14 day’ samples. Plasmid and gDNA materials were quantified by BioMark dPCR, in parallel with control samples stored at -20 °C. Storage of the replicate vials of BCG/ASM material commenced on three consecutive days, while all replicate vials of the plasmid and gDNA material were stored on the same day.

Genomic DNA was extracted from each BCG/ASM sample along with reference samples stored at -80 °C, using the CTAB/NaCl protocol. A single replicate set of samples (comprising of one reference sample and one vial for each of the storage conditions and durations) were extracted on three consecutive days. gDNA extracts were stored at -20 °C immediately after elution until quantification by BioMark dPCR.

**Table S1: dPCR Primers**

| Assay name | Oligonucleotide name | Oligo sequence | Reporter, Quencher | Amplicon length | BioMark [oligos] (nM) | QX100 [oligos] (nM) | QuantStudio™ 3D  [oligos] (nM) | qPCR efficiency (R^2^)* | Reference |
| --- | --- | --- | --- | --- | --- | --- | --- | --- | --- |
| UCL_16S | UCL_16S_F | GTGATCTGCCCTGCACTTC |  | 106 | 200 | 300 | 300 | 95%  (> 0.99) |  |
|  | UCL_16S_R | ATCCCACACCGCTAAAGCG |  |  | 200 | 300 | 300 |  | Honeyborne et al. [1] |
|  | UCL_16S_P | AGGACCACGGGATGCATGTCTTGT | [FAM]/NFQ |  | 200 | | 300 |  |  |
| GN_rpoB1 | RPOB_FW1 | CAAAACAGCCGCTAGTCCTAGTC |  | 84 | 900 | | 900 | 97%  (> 0.99) |  |
|  | RPOB_REV1 | AAGGAGACCCGGTTTGGC |  |  | 900 | | 900 |  | Devonshire et al. [2] |
|  | RPOB_PROBE1 | AGTCGCCCGCAAAGTTCCTCGAA | [FAM]/NFQ |  | 200 | | 300 |  |  |

Abbreviations: NFQ – non-fluorescent quencher. *rpoB* and 16S rRNA (alternative nomenclature: *rrs*) sequence information based on *M. tuberculosis* H37Rv, NC_018143 (regions 1471862..1473381 and 759810..763328, respectively).

* This value is reported in Devonshire et al. [2]

**Table S2: dPCR thermal cycling conditions**

| dPCR platform | Mastermix | Catalogue # | UNG incubation | PCR (temperature, time) | | | # Cycles | Droplet stabilisation/Final extension | Additional information |
| --- | --- | --- | --- | --- | --- | --- | --- | --- | --- |
|  |  |  |  | Enzyme activation | Temperature 1 | Temperature 2 |  |  |  |
| BioMark 37K qdPCR IFC | Taqman Gene Expression Master Mix (Life Technologies) | 4369016 | 50°C, 120s | 95°C, 600s | 95°C, 15s | 60°C, 60s | 40 | N/A | N/A |
| QX100 ddPCR System | 2X ddPCR Super Mix for probes (Bio-Rad) | 186-3010 | N/A | 95°C, 600s | 94°C, 30s | 60°C, 30s | 40 | 98°C, 600s (followed by maintenance at 4°C). | Ramp rates were restricted to 2.5 °C/s |
| QuantStudio™ 3D | QuantStudio™ 3D Digital PCR Master Mix | 4482710 for v1 | N/A | 96°C, 600s | 60°C, 60s | 96°C, 30s | 39 | 60^o^C, 120 sec | N/A |

**Table S3: dPCR partition volume, partition number and mean λ values**

| Template | dPCR platform | Partition volume (nL) | Mean $\lambda$ | Mean* total partition number |
| --- | --- | --- | --- | --- |
| Plasmid | BioMark | 0.85 | 1.12 | 770 |
| H37Rv | BioMark |  | 0.76 |  |
| BCG/ASM | BioMark |  | 1.01 |  |
| Total MTB Control | BioMark |  | 0.02 |  |
| Plasmid | QX100 | 0.83 | 1.07 | 14606 |
| H37Rv | QX100 |  | 0.79 | 14485 |
| BCG/ASM | QX100 |  | 0.94 | 14707 |
| Plasmid | QS3D | 0.809 | 0.90 | 18189 |
| H37Rv | QS3D |  | 0.65 | 18374 |

*Mean values per template type for QX100 (number of accepted droplets) and QS3D (following application of quality threshold). Partition number is fixed for BioMark.

C

B

A

**Figure S1: Stability testing of EQA materials.** Materials were stored at three conditions (dry ice, 4°C, 40°C) for 7 or 14 days (n = 3) prior to analysis and compared with control samples maintained at -20°C (Plasmid, H37Rv gDNA) or -80°C (BCG/ASM). Individual datapoints are displayed for each condition. Dotted lines indicate values assigned to materials from NMI inter-laboratory study (Table 1).

B

B

A

C

**Figure S2: Results of analysis of BCG/ASM and Total MTB Control materials by Gene Xpert MTB/RIF end-user laboratories.** The frequency of MTB detection levels from individual test results from all participating laboratories (n = 18) are shown for (A) BCG/ASM and (B) Total MTB Control materials. (C) Variation (SD) in Cq values for both EQA materials.

**Table S4: Extraction and qPCR methods of qPCR end-user laboratories**

| Laboratory name | Extraction method | Gene target (qPCR) | Reference (if applicable) |
| --- | --- | --- | --- |
| Borstel | DNA mini tissue kit (Qiagen) | CRISPR-associated protein Csm | Hillemann et al. [3, 4] |
| GOSH | DNA Mini kit (Qiagen), with an added bead-beating step | *pks-13, ESAT-6,*  *hupB, hsp65* | N/A |
| NUI Galway | IDI bead beating (3 minutes)  Quick gDNA™ MiniPrep (Zymo) | *lepA* | Reddington et al. [5] |

**Table S5: Xpert MTB/RIF assay and instrumentation information (Xpert end-user laboratories)**

| Laboratory name | Gene Xpert system version | MTB/RIF Assay version |
| --- | --- | --- |
| San Raffaele Scientific Institute | 4.4a | 5 |
| Lancet Laboratories | 6.1 | 5 |
| Forschungszentrum Borstel | 4.4a | 5 |
| UCL | 4.6a | 5 |
| TASK Applied Science | 4.4a | 5 |
| KCMC/KCRI | 6.1 | 5 |

**Table S6 : Cq (Ct) values from the difference instruments/ laboratories: single qPCR value and average XPERT MTB/RIF value from 5 probes. Laboratory β did not detect one of the replicate materials for the Total MTB Control. One of the Xpert MTB/RIF results from Laboratory 1 was omitted from the comparison (red cell) for the BCG/ASM analysis as it was a clear outlier result. This was further supported by a high SPC result of 30.6.**

|  | qPCR Laboratory | | | Xpert MTB/RIF Laboratory | | | | | |
| --- | --- | --- | --- | --- | --- | --- | --- | --- | --- |
| Material | α | β | γ | 1 | 2 | 3 | 4 | 5 | 6 |
| Total MTB Control | 32 |  | 36 | 26 | 25 | 24 | 22 | 24 | 24 |
|  | 31 | 35 | 36 | 25 | 26 | 26 | 24 | 23 | 26 |
|  | 32 | 36 | 36 | 26 | 25 | 24 | 24 | 22 | 24 |
| BCG/ASM | 21 | 26 | 26 | 19 | 21 | 19 | 16 | 21 | 19 |
|  | 20 | 28 | 28 | 18 | 23 | 20 | 20 | 20 | 17 |
|  | 20 | 26 | 29 | 26 | 20 | 22 | 19 | 21 | 20 |

References:

1. Honeyborne I, McHugh TD, Phillips PP, Bannoo S, Bateson A, Carroll N, Perrin FM, Ronacher K, Wright L, van Helden PD, Walzl G, Gillespie SH. Molecular bacterial load assay, a culture-free biomarker for rapid and accurate quantification of sputum *Mycobacterium tuberculosis* bacillary load during treatment. J Clin Microbiol. 2011;49:3905-11.
2. Devonshire AS, Honeyborne I, Gutteridge A, Whale AS, Nixon G, Wilson P, Jones G, McHugh TD, Foy CA, Huggett JF. Highly reproducible absolute quantification of *Mycobacterium tuberculosis* complex by digital PCR. Anal Chem. 2015;87:3706-13.
3. Hillemann D, Warren R, Kubica T, Rϋsch-Gerdes S, Niemann S. Rapid detection of *Mycobacterium tuberculosis* beijing genotype strains by real-time PCR. J Clin Microbiol. 2006;44:302-6.
4. Hillemann D, Warren R, Kubica T, Rϋsch-Gerdes S, Niemann S. Rapid detection of *Mycobacterium tuberculosis* beijing genotype strains by real-time PCR. J Clin Microbiol. 2006;44:3472.
5. Reddington K, Zumla A, Bates M, van Soolingen D,Niemann S, Barry T, O'Grady J. SeekTB, a two-stage multiplex real-time-PCR-based method for differentiation of the *Mycobacterium tuberculosis* complex. J Clin Microbiol. 2012;50:2203-6.
