# Supplementary material for: The use of digital PCR to improve the application of quantitative molecular diagnostic methods for tuberculosis
Source: BMC Infect Dis. 2016 Aug 3;16:366. doi: 10.1186/s12879-016-1696-7 (PMC4971652; doi:10.1186/s12879-016-1696-7)
Supplement: Additional file 2: — Protocol for gDNA extraction and dPCR quantification of materials. (PDF 394 kb) [file 12879_2016_1696_MOESM2_ESM.pdf]

EMRP
European Metrology Research Programme
Programme of EURAMET
The EMRP is jointly funded by the EMRP participating countries within EURAMET and the European Union

HLT08 INFECT-MET

Metrology for monitoring infectious diseases, antimicrobial resistance, and harmful micro-organisms

WP1 Inter-laboratory study:

Mycobacterium tuberculosis

Protocol

Task 1.7

(Assessment of performance of higher order measurement procedures for identification and quantification of infectious agents)

Coordinating laboratory:	LGC
Participating laboratories:	LGC, JRC, NIB

Contents

1. Introduction 4
  1.1 Background 4
  1.2 Aims 4
  1.3 Objectives 4
  1.4 Received materials and storage conditions 4
  1.5 Experimental design 5
2. Test materials 7
  2.1 Background 7
  2.2 TB Control Plasmid (nucleic acid molecule) 7
  2.3 H37Rv gDNA (nucleic acid extract) 7
  2.4 BCG in artificial sputum (whole microbe preparation) 7
  2.5 Test material stability 7
3. Protocol for gDNA extraction from BCG in artificial sputum 8
  3.1 Samples 8
  3.2 Equipment required 8
  3.3 Materials 8
  3.4 Reagents 8
  3.5 Notes 8
  3.6 Method 8
4. PCR assay information 10
  4.1 Oligonucleotide sequences and information 10
5. dPCR analysis 11
  5.1 dPCR experimental design 11
  5.2 Templates 11
  5.3 BioMark dPCR protocol 12
    5.3.1 Materials 12
    5.3.2 Reagents 12
    5.3.3 Reaction mix preparation 12

5.3.4 Instrument set-up 13

5.3.5 BioMark data analysis 13

5.4 QX100 droplet dPCR protocol 14

5.4.1 Materials 14

5.4.2 Reagents 14

5.4.3 Reaction mix preparation 14

5.4.4 Instrument set-up 15

5.4.5 Data analysis 15

6. Sample preparation for dPCR analysis 16

6.1 Sample nomenclature 16

6.2 Preparation of test sample aliquots 16

6.3 Sample Diluent preparation 16

6.4 Test sample dilution for dPCR 16

6.4.1 TB control plasmid 16

6.4.2 H37Rv gDNA 17

6.4.3 gDNA extracted from BCG in artificial sputum 17

7. Preparation of CTAB extraction reagents 18

7.1 CTAB/NaCl solution 18

7.2 NaCl (5M) 18

7.3 Chloroform/ Isoamyl alchohol 18

8. References 19

1. Introduction

1.1 Background

Test materials of three levels of complexity have been developed as prototype reference materials for *Mycobacterium tuberculosis* (*M. tuberculosis*) (D1.3.1): TB Control Plasmid (Level 1: Nucleic acid molecule); H37Rv genomic DNA (gDNA) (Level 2: Nucleic acid extract) and heat-inactivated *Mycobacterium bovis Bacillus Calmette–Guérin* (BCG*)* in artificial sputum "BCG in artificial sputum" (Level 3: Whole microbe material). BCG in artificial sputum test material requires extraction of gDNA prior to analysis using the Cetyltrimethylammonium bromide (CTAB) NaCl method (Soolingen and de Haas, 2001) which was found to be the optimum method in a comparison of different extraction approaches (D1.2.2 and D1.2.3).

Potential higher order amplification-based methods for quantification of *M. tuberculosis* genome copies have been developed using PCR assays which are specific to two *M. tuberculosis* target genes: 16S ribosomal RNA (16S rRNA) and RNA polymerase beta sub-unit (*rpoB*). These assays have been optimised for real-time quantitative PCR (qPCR) and digital PCR (dPCR) analysis (D1.5.2).

1.2 Aims

The aims of this inter-laboratory study are to assign values to test materials (plasmid or genome copies/µL) and to evaluate the reproducibility of potential higher order methods for tuberculosis (TB) quantification using dPCR.

1.3 Objectives

- Measurement of 16S rRNA and rpoB DNA copy numbers, in order to calculate the concentration of plasmid and TB genomic copies present in the three test materials.
- Evaluation of the variability of measurements performed at each test laboratory in order to gather information on the repeatability and intermediate precision of the potential higher methods.
- Evaluation of the comparability of dPCR measurements performed using two different platforms: (i) qdPCR 37k integrated fluidic circuit (IFC) arrays and BioMark PCR System (Fluidigm) (Section 5.3) and (ii) QX100 or QX200 Droplet Digital PCR System (Bio-Rad) (Section 5.4).
- Comparison of measurements performed at three different laboratories in order to evaluate the reproducibility of measurements.

1.4 Received materials and storage conditions

The below listed materials are distributed to participants:

- TB Control Plasmid (4 tubes)
- H37Rv gDNA (4 tubes)
- BCG in artificial sputum (4 tubes)
- 20×PP mixes (3 tubes):
    - UCL_16S (primers for BioMark) (100 µL)
    - UCL_16S (primers for QX100/QX200) (200 µL)
    - GN_rpoB1 (300 µL)
- Sample Diluent (1 tube)

Three units (tubes) of each test material are supplied to each laboratory for performing the study. An additional unit of each material is supplied for preliminary testing of methods. 16S rRNA and *rpoB* PCR assays (Section 4) are supplied as 20× solutions of primers and hydrolysis probe (“20×PP”). Sample Diluent consisting of sonicated human gDNA (Cambio, Cat# CA-972-05) is provided as a concentrated stock (500 ng/µL, 500 µL) for preparation of working stocks (Section 6).

Upon receipt of samples, TB Control Plasmid and H37Rv gDNA test materials, 20× PP solutions and Sample Diluent stock solution are to be stored at -20°C. BCG in artificial sputum should be stored at -80°C if available (if a -80°C freezer is not available, this material should be stored at -20°C).

1.5 Experimental design

It is proposed that three independent analyses consisting of dilution (Section 6) and dPCR (Section 5) of each unit of test material (“test sample”) are to be performed for each dPCR platform (a total of six experiments) (Figure 1) Each dPCR experiment consists of measurement of 16S rRNA and *rpoB* copy numbers in each test sample with two replicate reactions. It is recommended that a minimum of six aliquots of each test sample are prepared initially in order to standardise the number of freeze-thaw cycles prior to each experiment.

EMRP
European Metrology Research Programme
Programme of EURAMET
The EMRP is jointly funded by the EMRP participating countries within EURAMET and the European Union

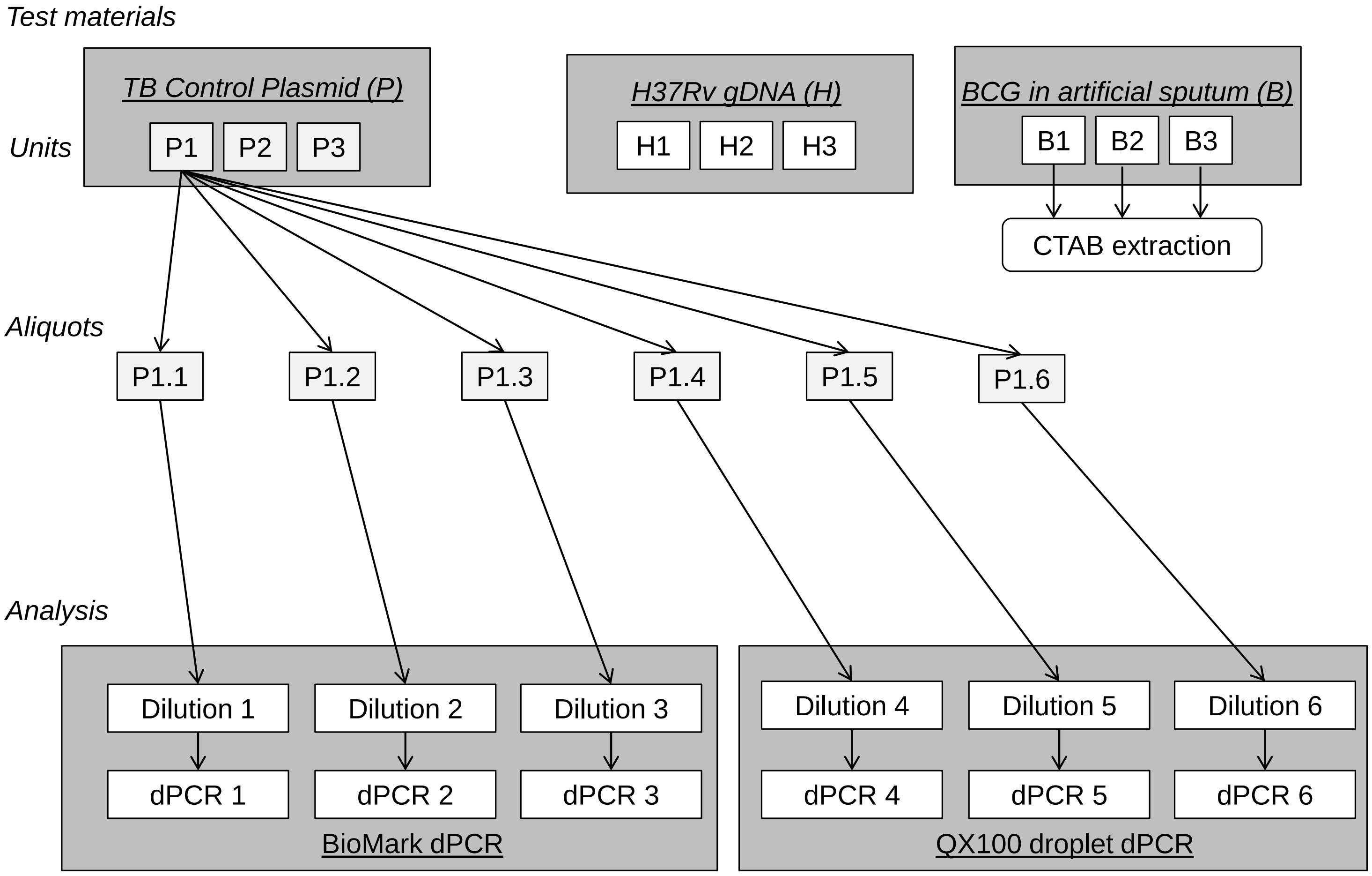

Figure 1: Experimental design of TB test material analysis

2. Test materials

2.1 Background

Please see the document Deliverable D1.3.1 for further information on preparation and contents of test materials. Concentrations of TB Control Plasmid and H37Rv gDNA were initially determined by Qubit® (fluorometric dsDNA High Sensitivity binding assay) analysis (D1.3.1), however further information based on preliminary qPCR or dPCR analysis (BioMark platform) of the test materials is provided here in order to ensure that samples are analysed within the optimum range for dPCR analysis.

2.2 TB Control Plasmid (nucleic acid molecule)

Restriction digestion of 100 ng TB Control Plasmid was performed with *Hind*III and the linearised plasmid was diluted to a concentration of ~10^5 copies/µL in a background of sonicated human gDNA (25 ng/µL). Preliminary dPCR analysis estimated the mean concentration of plasmid DNA to be approximately 1.4×10^5 copies/µL.

2.3 H37Rv gDNA (nucleic acid extract)

M. tuberculosis H37Rv gDNA obtained from ATCC was diluted in sonicated human gDNA (25 ng/µL) to a concentration of ~10^5 copies/µL. Initial dPCR analysis indicated this material to have a concentration of approximately 6.8×10^4 copies/µL.

2.4 BCG in artificial sputum (whole microbe preparation)

M. bovis BCG was grown in liquid culture and a suspension of mycobacteria spiked into artificial sputum matrix (ASM) in 1 mL aliquots. The number of colony-forming units (CFU) per mL of ASM was ascertained by solid agar counting and a mean value (± 95% confidence interval) of 1.1×10^7 ($\pm 1.3 \times 10^6$) CFU/mL ASM obtained. gDNA was extracted from ten sample units using the CTAB method (Section 3) and analysed for *rpoB* copy number by qPCR and dPCR. The average concentration was 1.3×10^7 genomic copies/unit with an inter-vial CV of 13%.

2.5 Test material stability

Suitability of the BCG in artificial sputum material for shipment was confirmed by performing a short-term stability study. Units of the test material (n = 3) were incubated on dry ice, +4°C and +40°C for 7 and 14 days compared to storage at the reference temperature of -80°C. gDNA was extracted from test units using CTAB NaCl (Section 3) and *rpoB* genomic equivalents measured by qPCR using an H37Rv gDNA standard curve. Analysis of variance (ANOVA) indicated no statistically significant differences between treatments ($p > 0.05$).

3. Protocol for gDNA extraction from BCG in artificial sputum

3.1 Samples

- BCG in artificial sputum test material (Section 2)

3.2 Equipment required

- Biosafety cabinet
- Fume hood
- Centrifuge, max speed > 10,000 g
- Water bath/heating block at 37°C
- Water bath at 65°C

3.3 Materials

- 1% Virkon (or similar virucidal disinfectant)

3.4 Reagents

- 10 mg/mL lysozyme (such as Sigma, Cat# L3790-10X1ML)
- CTAB/NaCl buffer (700 mM NaCl, 10% w/v CTAB, see Appendix I)
- SDS (10% in distilled H_2O, e.g. Sigma: 71736)
- Proteinase K (20 mg/mL)
- NaCl (5M, see Appendix I)
- Chloroform/Isoamyl alchohol (24:1 v/v, see Appendix I)
- Isopropanol (Molecular biology grade, >99.5% v/v, stored at < -20°C)
- Ethanol (70% v/v in distilled H_2O, stored at < -20°C)
- 1× Tris-EDTA (TE) solution

3.5 Notes

Samples should not be vortexed at any point during the extraction, as this could shear the gDNA. Aliquots of reagents required in the biosafety cabinet should be prepared, with the remainder discarded after use.

3.6 Method

Note: The required volumes (+ dead volume) of reagents marked with * (below) should be aliquotted into clean vials for use in the biosafety cabinet. Any remaining reagent in the aliquot should be discarded.

1. Thaw the required number of vials of frozen, heat killed BCG in artificial sputum. Briefly centrifuge vials and transfer to a biosafety cabinet.
2. Add 50 µL of 10 mg/mL lysozyme* and mix gently by stirring with the pipette.
3. Wipe vials with 1% Virkon before removing from the biosafety cabinet, and incubate overnight in a water bath (or heating block) set at 37°C.
4. Return the vials to the biosafety cabinet and set the water bath to 65°C. Pre-warm the CTAB/NaCl buffer to 65°C.
5. Add 70 µL 10% SDS* and 5 µL 20 mg/mL proteinase K* to each sample vial. Mix gently by stirring with the pipette tip.
6. Wipe vials with 1% Virkon, then relocate to the water bath and incubate at 65°C for 10 minutes.
7. Spin vials briefly and return to the biosafety cabinet.
8. Add 100 µL 5M NaCl* to each vial, followed by 100 µL of pre-warmed CTAB/NaCl buffer*. Mix gently with the pipette.
9. Wipe vials with 1% Virkon, remove to the water bath and incubate at 65°C for 10 minutes.
10. Spin vials briefly and relocate to a fume hood.
11. Add 750 µL of chloroform/isoamyl alcohol (24:1 v/v). Mix by inverting the tubes at least 10 times.
12. Remove samples from the fume hood and spin vials at 10,000 g for 5 minutes.
13. Appropriately label sterile, nuclease-free 1.5 mL vials and aliquot 450 µL of ice cold (-20°C) 100% Isopropanol to each.
14. In the fume hood, carefully transfer upper (aqueous) supernatant layers from step 12 into the appropriately labelled vials prepared in step 13. Take care not to disturb the aqueous/organic interface when removing supernatant. Discard organic layers.
15. Chill sample at -20 °C for 30 minutes.
16. Spin samples at 10,000 g for 15 minutes at ambient temperature.
17. Remove and discard the supernatants and wash pellets with 1 mL of ice-cold (-20°C) 70% Ethanol. Invert gently.
18. Spin vials at 10,000 g for 5 minutes at ambient temperature. Remove and discard as much ethanol as possible without disturbing the pellets.
19. Dry pellets by incubating with the lids open, in a heating block set at 65°C, until all of the ethanol has evaporated.
20. Rehydrate pellets in 200 µL TE buffer overnight at 4°C.
21. Prepare aliquots of gDNA extracted from BCG in artificial sputum and store extracts at -20°C.

4. PCR assay information

4.1 Oligonucleotide sequences and information

Participating laboratories are provided with a 20×PP mix containing primers and probe for the two assays to be used for the inter-laboratory study. Assays *UCL_16S* (Table 1) and *GN_rpoB1* (Table 2) are to be used to measure copy number of the TB target genes 16S rRNA and *rpoB* respectively. Further information is contained in D1.5.2 "Measurement procedures for detection and quantification of the model system".

Table 1: UCL_16S assay information

Primer/probe name	Sequence (5' - 3')	Length	Tm (°C)	Label	Amplicon size (bp)	Reference
UCL_16S_F	GTGATCTGCCCTGCACTTC	19	63.6		106	Honeyborne *et al.*, 2011
UCL_16S_R	ATCCCACACCGCTAAAGCG	19	67.2			
UCL_16S_P	AGGACCACGGGATGCATGTCTTGT	24	73.4	FAM/BHQ1		

Table 2: GN_rpoB1 assay information

Primer/probe name	Sequence (5' - 3')	Length	Tm (°C)	Label	Amplicon size (bp)	Reference
RPOB_FW1	CAAAACAGCCGCTAGTCCTAGTC	23	65		84	In-house assay
RPOB_REV1	AAGGAGACCCGGTTTGGC	18	66.3			
RPOB_PROBE1	AGTCGCCCGCAAAGTTCCTCGAA	23	75.1	FAM/BHQ1		

It is recommended that single-use aliquots of the 20×PP mixes are prepared in order to multiple freeze-thaw cycles of the reagents.

EMRP
European Metrology Research Programme
Programme of EURAMET
The EMRP is jointly funded by the EMRP participating countries within EURAMET and the European Union

5. dPCR analysis

5.1 dPCR experimental design

Each experiment (Figure 2) consists of independent dilution and dPCR analysis of all three units of each test material (a total of nine test samples). For each experiment, 36 reactions are required for dPCR analysis of all test samples with duplicate assays to 16S rDNA and *rpoB*. The remaining 12 panels of each qdPCR 37k array (BioMark) or ddPCR 96-well plate (QX100/200), respectively, should consist of no template controls (NTC) reactions: (1) Sample diluent (sonicated human genomic DNA, 25 ng/µL) (*n* = 3 for each assay) and (2) nuclease-free water (*n* = 3 for each assay).

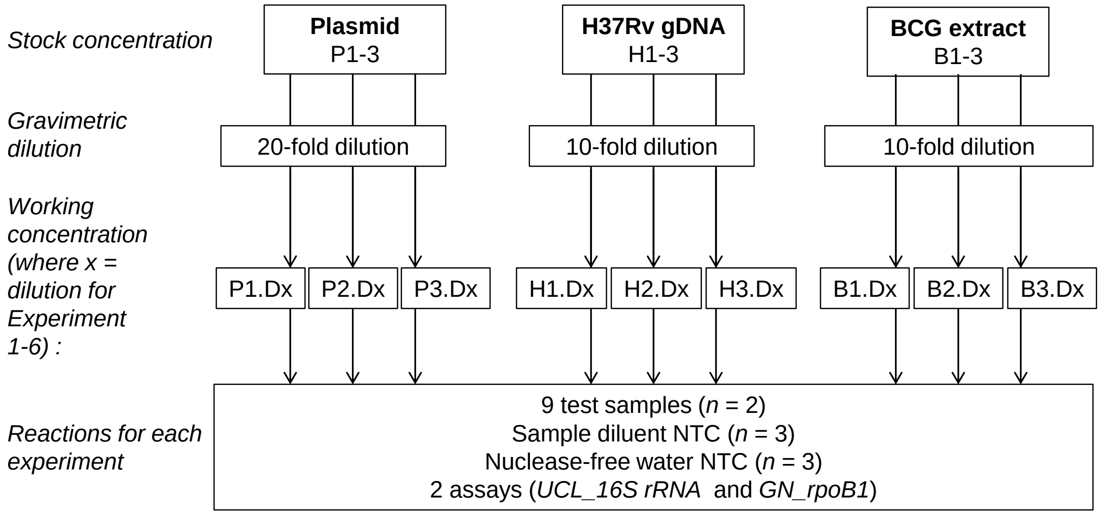

Figure 2: dPCR experimental design

Note: The UCL_16S assay was validated using a final concentration of 200 nM for the BioMark platform whereas a final primer concentration of 300 nM was found to be optimal for the QX100. Therefore separate 20×PP mixes are provided for each platform.

5.2 Templates

It is recommended that template dilution (Section 6) is performed after PCR mastermix preparation in order to avoid contamination of reaction mastermixes.

Following dilution, test samples are estimated to contain the following concentrations based on preliminary analysis of *rpoB* copy number. The concentrations have been chosen in order to result in a dPCR λ value of ~ 1.0.

- Diluted TB control plasmid (7.0×10^3 copies/µL)

EMRP
European Metrology Research Programme
▸ Programme of EURAMET
The EMRP is jointly funded by the EMRP participating countries within EURAMET and the European Union

- Diluted H37Rv gDNA (6.8 × 10^3 genome copies/µL)
- Diluted gDNA extracted from BCG in artificial sputum (6.5 × 10^3 copies/unit)

5.3 BioMark dPCR protocol

5.3.1 Materials

- qdPCR 37K IFC (Integrated Fluidic Circuit; Fluidigm, Cat# 100-6152)
- Control Line Fluid Kit (Fluidigm, Cat# 89000020)

5.3.2 Reagents

- Gene Expression Master Mix (Applied Biosystems, Cat# 4369016)
- GE Sample Loading Reagent (Fluidigm, Cat# 8500746)
- Nuclease-free water (such as Ambion, Cat# AM9937)
- Sample Diluent: Sonicated human genomic DNA (25 ng/µL, prepared from stock solution)
- UCL_16S assay 20×PP mix BIOMARK ONLY (provided)
- GN_rpoB1 assay 20×PP mix (provided)

5.3.3 Reaction mix preparation

For each panel, a 10 µL reaction is prepared to allow sufficient excess for pipetting 6 µL into each sample inlet and to avoid inclusion of air bubbles into the microfluidic circuit.

Tables 3 and 4 describe the composition of a single dPCR assay and recommended scaled volumes for preparation of a reaction mastermix sufficient for each experiment. Further details for 16S rDNA and *rpoB* assays are included in Section 4.

Table 3: BioMark dPCR assay and mastermix composition (UCL_16S assay)

Component	[Final]	Volume 1 reaction (µL)	Volume ½ array (30 reactions) (µL)
Gene Expression Master Mix	1×	5.0	150.0
Sample Loading Reagent	2×	1.0	30.0
Forward primer	200 nM	0.5 (20× PP)	15.0 (20× PP)
Reverse primer	200 nM		
Probe [FAM/NFQ]	200 nM		
Nuclease-free water	N/A	1.5	45.0
DNA	N/A	2.0	
	Total (µL)	10.0	210.0

Table 4: BioMark dPCR assay and mastermix composition (GN_rpoB1 assay)

Component	[Final]	Volume 1 reaction (µL)	Volume ½ array (30 reactions) (µL)
Gene Expression Master Mix	1×	5.0	150.0
Sample Loading Reagent	2×	1.0	30.0
Forward primer	900 nM	0.5 (20× PP)	15.0 (20× PP)
Reverse primer	900 nM		
Probe [FAM/NFQ]	200 nM		
Nuclease-free water	N/A	1.5	45.0
DNA	N/A	2.0	
	Total (µL)	10.0	210.0

Replicate reactions (*n* = 2) are to be prepared for each diluted test sample. For recommended dilution factors see Section 6.

5.3.4 Instrument set-up

Priming and loading of qdPCR 37K IFCs are to be performed as per manufacturer's standard protocol. The following information is to be specified during dPCR set-up using BioMark Data Collection software:

- Detector to be selected as 'Single Probe' and 'FAM-MGB'.
- Thermal cycling conditions (Table 5): '10min-Hot-start 40 cycles' (default protocol)

Table 5: BioMark dPCR thermal cycling conditions

Cycles	Time	Temp (°C)
1	2 min	50
1	10 min	95
40	15 sec	95
	60 sec	60

5.3.5 BioMark data analysis

It is recommended that positive amplifications are counted between PCR cycles 20 and 40 with manual Cq threshold setting for each assay ("Detector") and linear baseline correction. Please report Cq threshold, quality threshold, positive amplifications per panel ("Count") and copy number per panel ("Estimated targets") from each experiment in the data submission spreadsheet.

5.4 QX100 droplet dPCR protocol

5.4.1 Materials

- DG8 cartridge (Bio-Rad, Cat# 186-4008)
- Droplet generator oil (Bio-Rad, Cat# 290-10430)
- Pierceable Foil Heat Seal (Bio-Rad, Cat# 181-4040)

5.4.2 Reagents

- ddPCR Supermix for Probes (Bio-Rad, Cat# 186-3010)
- Nuclease-free water (such as Ambion, Cat# AM9937)
- Sample Diluent: Sonicated human genomic DNA (25 ng/µL, see Section 6)
- UCL_16S assay 20×PP mix BIORAD ONLY (provided)
- GN_rpoB1 assay 20×PP mix (provided)

5.4.3 Reaction mix preparation

Table 6 and Table 7 describe the composition of a single droplet dPCR assay and recommended scaled volumes for preparation of reaction mastermix sufficient for each experiment. Further details for 16S rDNA and *rpoB* assays are included in Section 4.

Table 6: QX100 dPCR assay and mastermix composition (UCL_16S assay)

Component	[Final]	Volume 1 reaction (µL)	Volume 30 reactions (µL)
ddPCR Supermix	1×	10.0	300.0
Forward primer	**300 nM**	1.0 (20× PP mix)	30.0 (20× PP mix)
Reverse primer	**300 nM**		
Probe [FAM/NFQ]	200 nM		
Nuclease-free water	N/A	5.0	150.0
DNA	N/A	4.0	
	Total (µL)	20.0	420.0

Table 7: QX100 dPCR assay and mastermix composition (GN_rpoB1 assay)

Component	[Final]	Volume 1 reaction (µL)	Volume 30 reactions (µL)
ddPCR Supermix	1×	10.0	300.0
Forward primer	900 nM	1.0 (20× PP mix)	30.0 (20× PP mix)
Reverse primer	900 nM		
Probe [FAM/NFQ]	200 nM		
Nuclease-free water	N/A	5.0	150.0
DNA	N/A	4.0	
	Total (µL)	20.0	420.0

Typically, individual reactions containing both PCR mastermix and sample template were prepared to include a 10% excess (total volume 22 µL/reaction) to allow for pipetting 20 µL into the DG8 cartridge. Droplet generation should be performed using the DG according to the manufacturer's standard procedure. 40 µL of droplets was pipetted into wells of a 96-well plate and was sealed with a foil sheet using the BioRad PX1 PCR plate sealer for 6 seconds at 170°C.

Replicate reactions (*n* = 2) are to be prepared for each diluted test sample. For recommended dilution factors see Section 6.

5.4.4 Instrument set-up

PCR was performed by the coordinating laboratory using a C1000 Touch Thermal Cycler (Bio-Rad) using the cycling conditions below (Table 8):

Table 8: Droplet dPCR thermal cycling conditions

Step	# of cycles	Time	Temp (°C)
Enzyme activation	1	10 min	95
Denaturation<br>Annealing/extension*	40	30 sec<br>1 min	94<br>60
Enzyme Heat Kill	1	10 min	98
Hold (optional)	1	∞	4

* Ramp Rate settings to ~2.5 ^{o}C/sec. Heated lid: 105 °C

5.4.5 Data analysis

For each reaction, please report number of positive droplets and total number of accepted droplets from each experiment in the data submission spreadsheet.

EMRP
European Metrology Research Programme
Programme of EURAMET
The EMRP is jointly funded by the EMRP participating countries within EURAMET and the European Union

6. Sample preparation for dPCR analysis

6.1 Sample nomenclature

Test materials are given the following alphabetical abbreviations (Figures 1 and 2): TB Control Plasmid (P); H37Rv gDNA (H) and BCG in artificial sputum (B). Each unit of test material is designated numerically: for example, TB control plasmid unit 1 is given the nomenclature P1. A dilution of the test sample for Experiment x is designated P1.Dx (e.g. dilution of sample P1 for Experiment is designated P1.D1).

6.2 Preparation of test sample aliquots

Each dPCR experiment consists of an independent dilution prior to dPCR. Therefore, aliquots of each TB control plasmid (P), H37Rv gDNA (H) test sample and gDNA extracted from BCG in artificial sputum (B) should be prepared prior to the first experiment and re-frozen at -20µC. For example, six aliquots of 6 µL could be prepared to allow 5 µL to be used for dilution for each dPCR experiment. Aliquots can be designated P1.1-P1.6 (Figure 1).

6.3 Sample Diluent preparation

A working stock of sample diluent (25 ng/µL) should be prepared by 20-fold dilution of the stock solution (500 ng/µL) with nuclease-free water. Approximately 700 µL of Sample Diluent (working solution) is required for each experiment for test sample dilution and dPCR NTCs (n = 3 per assay; n = 6 per experiment).

6.4 Test sample dilution for dPCR

All test samples require dilution prior to dPCR analysis. Dilution should be performed gravimetrically. Test sample dilution should be performed on the same day as dPCR analysis. Dilution factors are the same for both BioMark dPCR (designated Experiments 1-3, Figure 1) (Section 5.3) and QX100/200 droplet dPCR (designated Experiments 4-6, Figure 1) (Section 5.4) analysis. Please note the template nomenclature used in the submission spreadsheets.

Tables for recording mass measurements are available in the data submission spreadsheet. An instrument accurate to 0.001 g should be used. It is recommended that test samples are mixed with Sample Diluent by brief (~1 second) vortexing to ensure that diluted templates are thoroughly homogenised before addition to reaction mixes.

6.4.1 TB control plasmid

TB plasmid at the provided stock concentration should be diluted in Sample Diluent by a factor of 20 by mixing 5 µL of test material with 95 µL of diluent.

6.4.2 H37Rv gDNA

H37Rv at the provided stock concentration should be diluted in Sample Diluent by a factor of 10 by mixing 5 µL of test material in 45 µL of diluent.

6.4.3 gDNA extracted from BCG in artificial sputum

It is recommended that gDNA extracted from BCG in artificial sputum using the protocol provided in Section 3 is diluted 10 fold prior to dPCR by mixing 5 µL with 45 µL Sample Diluent.

EMRP
European Metrology Research Programme
Programme of EURAMET
The EMRP is jointly funded by the EMRP participating countries within EURAMET and the European Union

7. Preparation of CTAB extraction reagents

7.1 CTAB/NaCl solution

Dissolve 4.1 g of NaCl in 20 mL of distilled H_2O. Add 10 g CTAB (Cetyltrimethylammonium bromide 99+%) and heat to 65 °C until dissolved. Make up volume to 100 mL with distilled water and autoclave at 121 °C for 20 minutes. Store at 4 °C for up to 6 months.

7.2 NaCl (5M)

Add 5.84 g of NaCl to a 50 mL Duran bottle, or equivalent. Make up volume to 20 mL with distilled water. Heat to 65 °C and mix until dissolved (this may take hours). Autoclave at 121 °C for 20 minutes. Store at ambient temperature for up to 12 months. **Note:** This method may not scale up effectively, as the NaCl does not dissolve completely. If more volume is required, multiply the preparations and pool before autoclaving.

7.3 Chloroform/ Isoamyl alcohol

To 48 mL of Chloroform (ACS reagent grade, >99.8%), add 2 mL of Isoamyl alcohol (GLC, 98+ %). Mix thoroughly by shaking vigorously for 5 seconds.

8. References

Honeyborne I, McHugh TD, Phillips PPJ, Bannoo S, Bateson A, Carroll N, Perrin FM, Ronacher K, Wright L, van Helden PD, Walzl G, Gillespie SH. 2011. Molecular Bacterial Load Assay, a Culture-Free Biomarker for Rapid and Accurate Quantification of Sputum *Mycobacterium tuberculosis* Bacillary Load during Treatment. *Journal of Clinical Microbiology* 49:3905-3911.

Soolingen, D., de Haas, P.E. (2001). Chapter 12: Restriction Fragment Length Polymorphism Typing of Mycobacteria; *Mycobacterium tuberculosis* protocols, edited by T. Parish and N.G. Stoker, ISBN 0-89603-776-2.
